# A case series of 15 patients with oesophageal cancer in Blantyre, Malawi. Risk factors for oesophageal squamous cell carcinoma in Sub-Saharan Africa

**DOI:** 10.1186/1753-6561-6-S4-P46

**Published:** 2012-07-09

**Authors:** S Granger

**Affiliations:** 1 University of Liverpool, UK

## Introduction

Oesophageal cancer rates are increasing in Sub-Saharan Africa: the vast majority of cases are oesophageal squamous cell carcinoma (OSCC). Alcohol and tobacco use are significant risk factors for the development of OSCC in developed countries but are less significant in developing countries like Malawi where other factors are implicated.

## Aim

To review exposure to risk factors in patients with OSCC in Malawi.

## Methods

A case series of 15 patients with oesophageal cancer from Queen Elizabeth Central Hospital, Blantyre, Malawi was collected from Queen Elizabeth Central Hospital endoscopy unit over a 4 week study period. All patients completed a questionnaire on known and potential risk factors for OSCC.

## Results

Alcohol and tobacco use were not significant risk factors in female patients; 1(12.5%) female reported 1 pack year history of smoking and none consumed alcohol. Smoking (71.5% of patients) and regular alcohol consumption (57.2% of patients) were common risk factors in men. Exposure to smoke from cooking on wood fires was common in females (87.5%) spending on average 3 hours cooking inside in poorly ventilated kitchens. All patients consumed a daily maize based diet, which has been implicated in the pathogenesis of oesophageal cancer via several mechanisms. Patients reported chronic consumption of boiling hot tea in 86.7% of cases and eating nsima (a local maize staple) when boiling hot in 66.7% of cases. Interestingly, two patients reported a family history of oesophageal cancer in their children.

## Conclusions

These findings support the role of alternative risk factors to smoking and alcohol use in the aetiology of OSCC in Malawi, particularly in females. A maize based diet, chronic exposure to smoke from cooking on wood fires and chronic consumption of boiling hot food and drink are all implicated as alternative factors. A multi-centre, case control study examining these risk factors is needed to support these findings so primary preventative measures can be developed against this devastating disease.

